# Multiple Rad52-Mediated Homology-Directed Repair Mechanisms Are Required to Prevent Telomere Attrition-Induced Senescence in *Saccharomyces cerevisiae*

**DOI:** 10.1371/journal.pgen.1006176

**Published:** 2016-07-18

**Authors:** Clémence Claussin, Michael Chang

**Affiliations:** European Research Institute for the Biology of Ageing, University of Groningen, University Medical Center Groningen, Groningen, The Netherlands; University of Montreal/IRIC, CANADA

## Abstract

Most human somatic cells express insufficient levels of telomerase, which can result in telomere shortening and eventually senescence, both of which are hallmarks of ageing. Homology-directed repair (HDR) is important for maintaining proper telomere function in yeast and mammals. In *Saccharomyces cerevisiae*, Rad52 is required for almost all HDR mechanisms, and telomerase-null cells senesce faster in the absence of Rad52. However, its role in preventing accelerated senescence has been unclear. In this study, we make use of *rad52* separation-of-function mutants to find that multiple Rad52-mediated HDR mechanisms are required to delay senescence, including break-induced replication and sister chromatid recombination. In addition, we show that misregulation of histone 3 lysine 56 acetylation, which is known to be defective in sister chromatid recombination, also causes accelerated senescence. We propose a model where Rad52 is needed to repair telomere attrition-induced replication stress.

## Author Summary

Telomeres are essential structures located at the ends of chromosomes. The canonical DNA replication machinery is unable to fully replicate DNA at chromosome ends, causing telomeres to shorten with every round of cell division. This shortening can be counteracted by an enzyme called telomerase, but in most human somatic cells, there is insufficient expression of telomerase to prevent telomere shortening. Cells with critically short telomeres can enter an arrested state known as senescence. Telomere attrition has been identified as a hallmark of human ageing. Homologous recombination proteins are important for proper telomere function in yeast and mammals. Yeast lacking both telomerase and Rad52, required for almost all recombination, exhibits accelerated senescence, yet no apparent increase in the rate of telomere shortening. In this study, we explore the role of Rad52 during senescence by taking advantage of *rad52* separation-of-function mutants. We find that Rad52 acts in multiple ways to overcome DNA replication problems at telomeres. Impediments to telomere replication can be dealt with by post-replication repair mechanisms, which use a newly synthesized sister chromatid as a template to replicate past the impediment, while telomere truncations, likely caused by the collapse of replication forks, can be extended by break-induced replication.

## Introduction

Telomeres, nucleoprotein structures located at the ends of linear chromosomes, prevent natural chromosome ends from being recognized as DNA double-strand breaks (DSBs) [1]. Due to incomplete DNA replication and nucleolytic degradation, telomeres shorten with each round of replication, which can eventually lead to a growth arrest, known as replicative senescence, or to apoptosis. Telomere shortening can be counteracted by a specialized reverse transcriptase called telomerase, which is composed of a protein catalytic subunit and an RNA subunit [2, 3]. Telomerase extends telomeres by iterative reverse transcription of a short sequence to the 3′ ends of telomeres, using the RNA subunit as a template [2, 4, 5].

Most human somatic cells do not express sufficient telomerase to prevent telomere shortening, which may be a contributing factor towards human ageing. This absence of telomere maintenance may have evolved as a barrier to tumorigenesis (reviewed in [6]). Indeed, cancer cells need to activate a telomere maintenance mechanism, and in approximately 85–90% of cancers, this occurs through the upregulation of telomerase [7]. The remaining 10–15% of cancers employ telomerase-independent, recombination-based mechanisms, collectively termed alternative lengthening of telomeres (ALT) [8].

ALT mechanisms were first described in the budding yeast *Saccharomyces cerevisiae*, where cells using ALT are called “survivors” [9]. There are two main types of survivors: type I and type II. Both types of survivors require the major recombination protein Rad52 and the DNA polymerase δ subunit Pol32 [9, 10]. Pol32 is essential for break-induced replication (BIR) [10], while Rad52 is important for almost all recombination-related activities, including BIR (reviewed in [11]). Type I survivors also require Rad51, Rad54, and Rad57, and maintain telomeres by amplification of subtelomeric Y′ elements [9, 12]. Formation of type II survivors, which exhibit amplification of the C_1–3_A/TG_1–3_ telomeric repeats, is Rad51-independent, but requires the MRX complex (consisting of Mre11, Rad50 and Xrs2), Rad59, and Sgs1 [12–15]. BIR can be Rad51-dependent or Rad51-independent, suggesting that type I and type II survivors maintain telomeres through Rad51-dependent BIR and Rad51-independent BIR, respectively [16, 17].

While recombination is clearly important for the maintenance of telomeres in survivors, recombination proteins are also important in pre-senescent cells [18]. Rad52 can be detected at telomeres well before the appearance of survivors [19]. Furthermore, telomerase-negative cells lacking Rad51, Rad52, Rad54, Rad57, Rad59, Pol32, or Sgs1 senesce very rapidly [9, 14, 15, 20–22]. With the exception of Sgs1, the enhanced senescence does not appear to cause a change in bulk telomere shortening [9, 12, 20, 23], although rare telomere loss events may be occurring. *tlc1Δ sgs1Δ* strains fail to resolve recombination intermediates at telomeres in pre-senescent cells, which may explain their accelerated senescence [24].

Rad52 mediates the exchange of RPA for Rad51 on single-stranded DNA to promote Rad51-catalyzed strand invasion [25, 26]. While this Rad51 pathway, which also requires Rad54, Rad55, and Rad57, is important for the majority of homology-directed repair (HDR), Rad52 also has Rad51-independent functions. These functions involve its DNA annealing activity, which is augmented by Rad59 [27–29]. The Rad51-mediator and the DNA annealing functions of Rad52 are separable. An alanine scan mutation study identified a class of *rad52* mutants (class C mutants) that can still promote recruitment of Rad51 but is deficient in DNA annealing [30, 31]. These mutants are defective in repairing DSBs and in sister chromatid recombination (SCR) but perform BIR with only slightly reduced efficiency [30, 32, 33].

The mechanism by which HDR prevents accelerated senescence has been poorly characterized. This is in part due to the multiple Rad52 subpathways within HDR. Rad51-dependent BIR and Rad51-independent BIR have been previously implicated in delaying senescence [21–23]. In this study, we make use of *rad52* class C mutants to show that SCR is also important during senescence. We also demonstrate that proper regulation of the acetylation of lysine 56 of histone 3 (H3K56) is important during replicative senescence, and we propose a model where Rad52 is repairing damage at telomeres in the absence of telomerase.

## Results

### Using telomere sequencing to assay recombination

**Fig 1:**
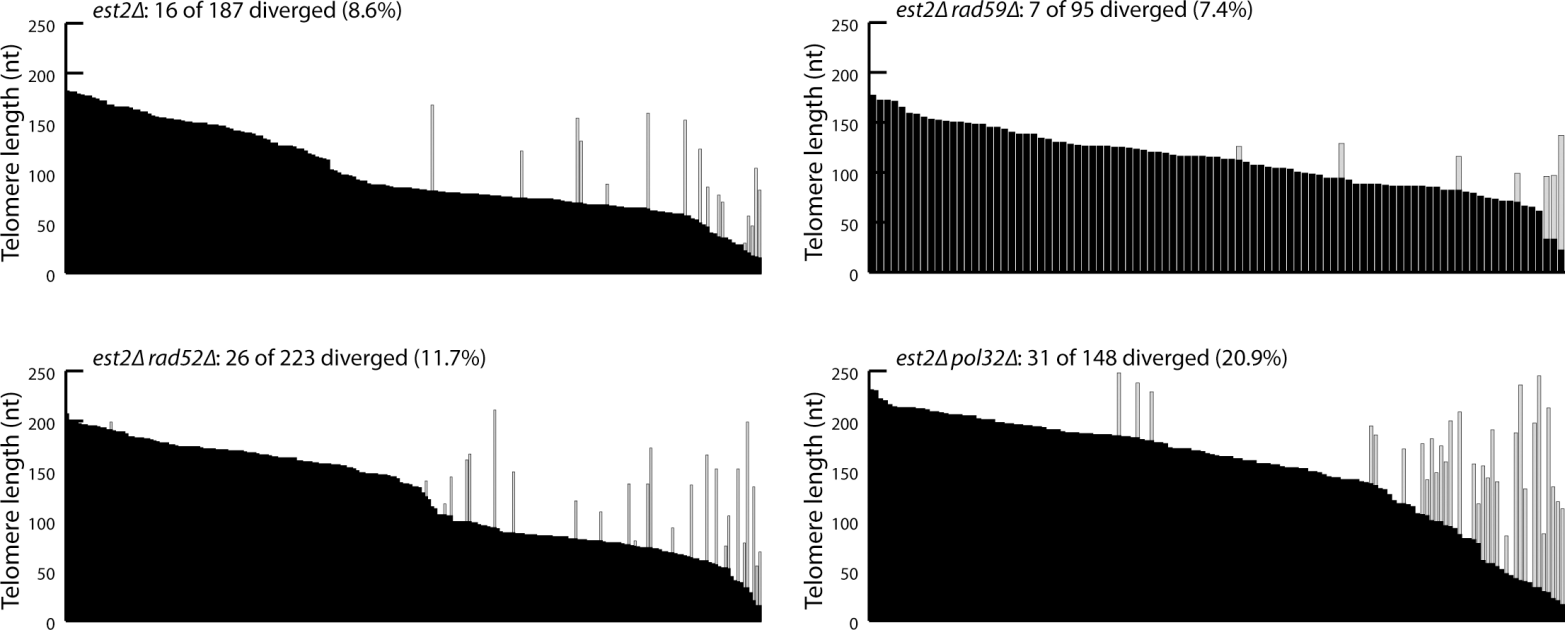
Telomere sequence divergence events in pre-senescent *est2Δ* cells are independent of Rad52. Telomere VI-R was amplified and sequenced from clonal populations of *est2Δ* (derived the sporulation of CCY16 and CCY8), *est2Δ rad52Δ* (derived the sporulation of CCY16), *est2Δ rad59Δ* (derived the sporulation of CCY8), and *est2Δ pol32Δ* cells (derived from the sporulation of CCY18), ~30 population doublings after isolation of haploid spores. Each bar represents an individual telomere and bars are sorted by the length of the undiverged sequence (black portion of each bar). The light gray portion of each bar represents the diverged region.

Previous studies have used telomere sequencing to detect recombination events in senescing *S*. *cerevisiae* cells [24, 34–42]. This assay takes advantage of the fact that yeast telomerase adds imperfect, degenerate repeats [43]. Sequencing multiple copies of the same telomere derived from a clonal population of cells reveals a centromere-proximal region of stable sequence and a distal region with differing degenerate repeats [44, 45]. The variation in the sequence of the distal region is largely abolished in the absence of telomerase [45], but rare sequence divergence events can be detected and have been presumed to be caused by recombination [34]. More precisely, since equal SCR generates repair products without changes in DNA sequence, the assay detects sequence divergence due to unequal SCR, intertelomere recombination, or BIR that does not result from perfect alignment with a sister telomere. These recombination events may be directly important in delaying senescence, or they may be a byproduct of other recombination-mediated activities that delay senescence. To determine the nature of these events, we sequenced telomere VI-R from *est2Δ* strains—*EST2* encodes the protein catalytic subunit of telomerase [3]—that are also deleted for either *RAD52*, *POL32*, or *RAD59*. All three of these genes are required for recombination of telomeric repeats in type II survivors [9, 10, 12]. In *est2Δ* cells, 8.6% of the telomeres exhibit sequence divergence, similar to what has previously been reported [37]. Surprisingly, even though *rad52Δ*, *pol32Δ*, and *rad59Δ* telomerase-null strains senesce rapidly [9, 21, 22], we find that the divergence events do not decrease in the absence of Rad52, Pol32, or Rad59 (Fig 1), indicating that these events are not involved in the recombination-mediated delay of senescence. In fact, divergence events increase in the absence of Pol32.

*pol32Δ rad52Δ* double mutants are synthetic lethal [46, 47]. One interpretation of this genetic interaction is that in the absence of Pol32, DNA replication is compromised, resulting in damage that is repaired by Rad52-dependent HDR. Indeed, we see elevated levels of Rad52 focus formation in *pol32Δ* cells (S1 Fig). The increased divergence seen in *est2Δ pol32Δ* telomere sequences could be due to an increase in Rad52-dependent recombination at telomeres. Consistent with this hypothesis, we observe a further increase in Rad52 focus formation in *est2Δ pol32Δ* cells.

To determine the source of the Rad52-independent divergence events, we performed two controls. First, we took two plasmids with cloned and sequenced telomeres (one of 166 bp and the other 213 bp in length), amplified the telomeres by PCR, re-cloned them into the same vector, and sequenced multiple clones. We found that 3.8% of the clones exhibited sequence divergence (S2 Fig). The divergence events can be due to sequence alterations caused during PCR amplification, propagation in bacteria, and/or DNA sequencing. Second, we integrated two telomeres, 166 bp and 230 bp in length, into the *URA3* locus in wild-type and *rad52Δ* strains, which were then clonally propagated for ~30 population doublings. We amplified these internal telomeres by PCR, cloned the PCR products, and sequenced multiple clones. We found that 4.2% and 7.3% of the clones from wild type and *rad52Δ*, respectively, exhibit sequence divergence (S2 Fig). The higher percentage in strains lacking Rad52 likely reflects a role for Rad52 in suppressing the accumulation of mutations [48]. While an internally-integrated telomere is not equivalent to a natural telomere, our data suggest that a significant fraction of sequence divergence events at natural telomeres in telomerase-null cells occur because of technical reasons related to amplification, cloning, and/or sequencing of telomeres. Our findings have implications with regard to using this assay to study recombination at telomeres (see Discussion), and show that the function of Rad52 in delaying senescence is unrelated to the sequence divergence events observed in senescing telomerase-negative cells. In addition, our data indicate that any Rad52-mediated HDR events during senescence most likely involves perfectly aligned sister telomeres, which would not alter the sequences of recombining telomeres, and would therefore not be detected using this assay.

### Rad52 is not preventing telomere truncation events

**Fig 2:**
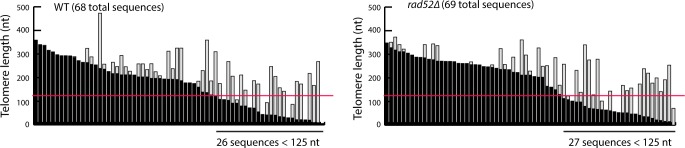
Deletion of *RAD52* does not increase telomere truncation events. Telomere VI-R was amplified and sequenced from clonal populations of wild-type and *rad52Δ* cells (derived from the sporulation of W8758), ~35 population doublings after the isolation of haploid spores. Each bar represents an individual telomere and bars are sorted by the length of the undiverged sequence (black portion of each bar). The light gray portion of each bar represents the diverged region. The red line highlights 125 nt. The number of telomeres with less than 125 nt of undiverged sequence is shown.

Although absence of Rad52 does not alter the rate of bulk telomere shortening, truncation of a small number of telomeres may be occurring that results in accelerated senescence. It was previously determined that telomeres less than 125 bp in length are highly unlikely to arise due to the standard end-replication problem [49, 50], which shorten telomeres by 3–4 bp per generation in yeast [51], so such telomeres would mostly likely have undergone a truncation event. To determine whether Rad52 prevents such truncation events, we sequenced telomeres from two wild-type and two *rad52Δ* telomerase-positive strains, which were derived from the meiosis of a single *rad52Δ*/*RAD52* diploid cell, after ~35 generations of clonal expansion. In telomerase-positive strains, most sequence divergence events are due to telomerase-mediated telomere extension, and not the telomerase- and Rad52-independent divergence events discussed above. The length of the undivergent region of each telomere indicates how short the telomere became before being extended by telomerase. It has previously been shown that telomeres with undivergent regions less than 125 bp in length do occur even in wild-type cells [49]. We confirm this observation and also find no change in the frequency of these truncation events in the absence of Rad52 (Fig 2). This suggests that Rad52 does not prevent telomere truncation events, although it may have a role in repairing such truncations.

### Loss of Rad52-mediated BIR does not fully account for the fast senescence of *est2Δ rad52Δ* mutants

**Fig 3:**
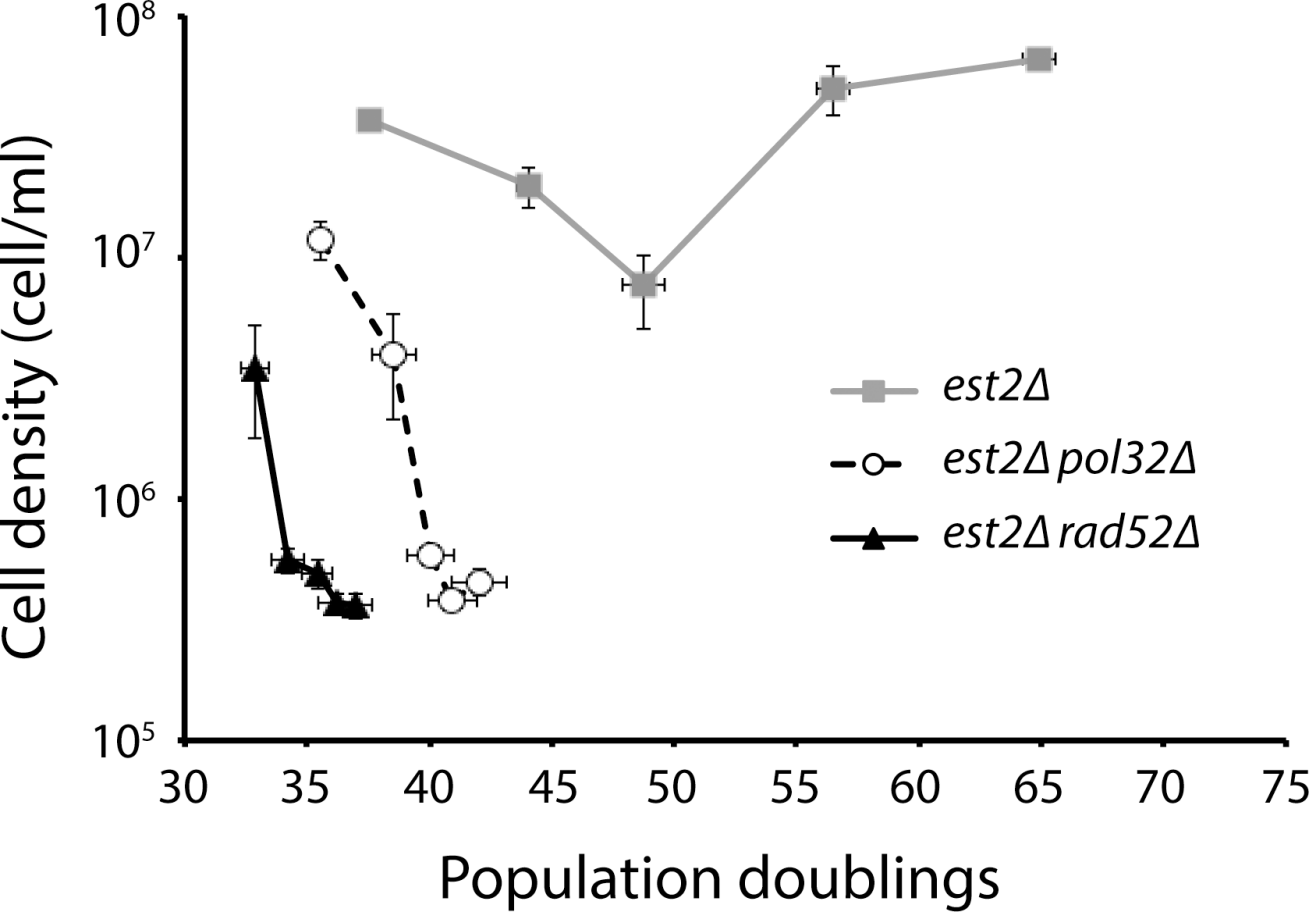
BIR does not fully account for the role of Rad52 in preventing accelerated senescence. Senescence rates were measured by serial passaging *est2Δ*, *est2Δ pol32Δ*, and *est2Δ rad52Δ* strains (derived from the sporulation of CCY155) in liquid culture. Cell density was measured each day after 24 h of growth in liquid culture, followed by dilution to 2 x 10^5^ cells/ml. Mean ± SE for five independent spore isolates for *est2*Δ and ten isolates for both *est2Δ pol32Δ* and *est2Δ rad52Δ* is shown.

Telomerase-negative strains lacking Pol32 have previously been shown to exhibit an accelerated rate of senescence [22], indicating the importance of BIR during senescence. Thus, the function of Rad52 in preventing accelerated senescence may be to promote repair of truncated telomeres through Pol32-mediated BIR. If the accelerated senescence of an *est2Δ rad52Δ* mutant is due to the role of Rad52 in BIR, then *est2Δ rad52Δ* and *est2Δ pol32Δ* mutants, derived from the same parental diploid, should have similar rates of senescence. Interestingly, we find that *est2Δ rad52Δ* mutants senesce faster than *est2Δ pol32Δ* mutants (Fig 3), indicating that although BIR is important to prevent accelerated senescence, other Rad52-mediated activities are also required.

### *est2Δ rad52* class C mutants exhibit accelerated senescence and an inability to form type II survivors

**Fig 4:**
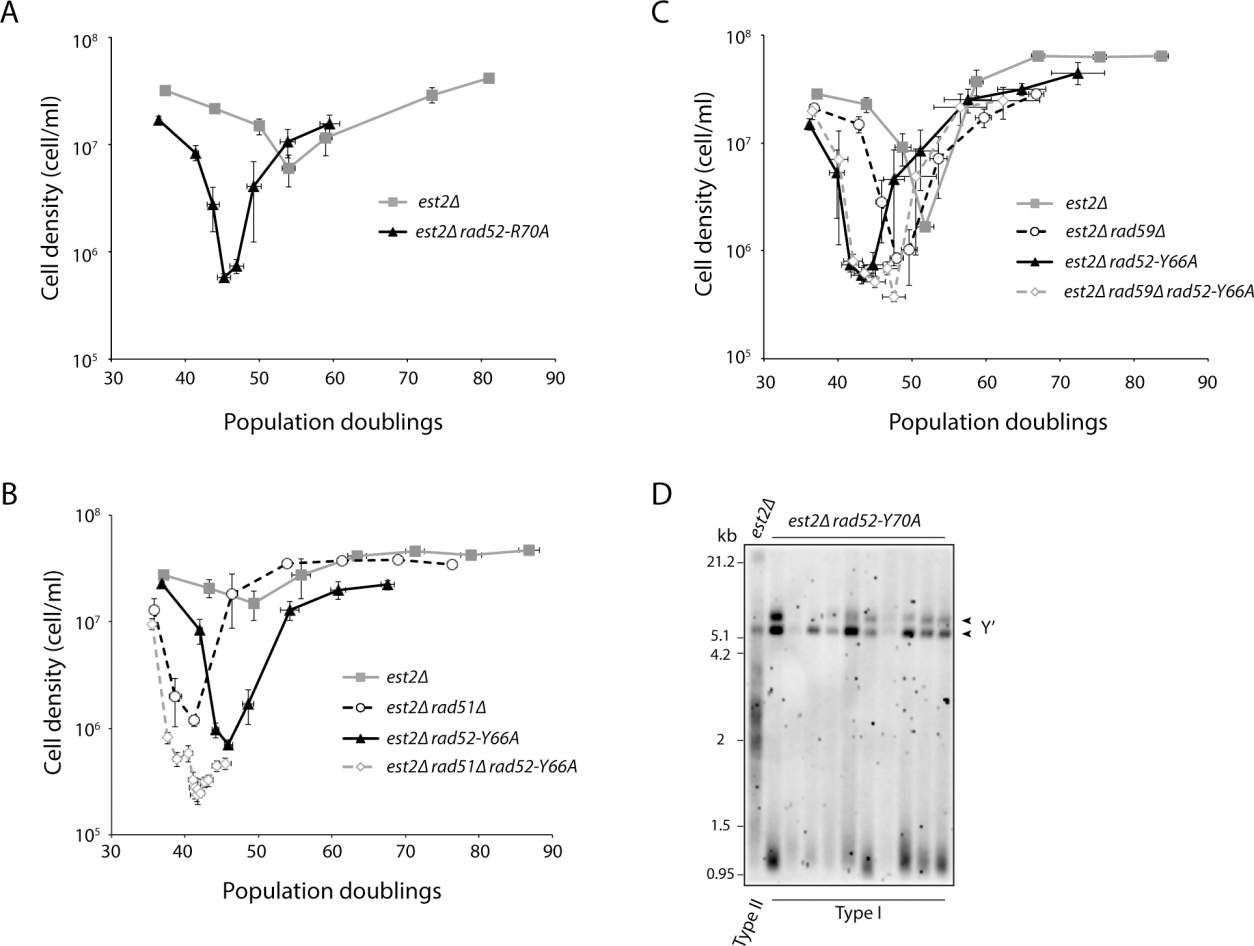
The strand annealing activity of Rad52 is important for preventing accelerated senescence and for the formation of type II survivors. (A–C) Senescence rates were measured in liquid culture by serial passaging of haploid meiotic progeny of the indicated genotypes, derived from the sporulation of CCY114 (A), CCY126 (B), or CCY127 (C). Cell density was measured each day after 24 h of growth in liquid culture, followed by dilution to 2 x 10^5^ cells/ml. Mean ± SE for each genotype is shown. At least eleven independent isolates for each genotype were followed in A, and at least four in B and C. (D) Telomere Southern blot of *est2Δ* and *est2Δ rad52-R70A* survivors obtained from the liquid culture senescence assay from A. *est2Δ rad52-R70A* survivors were analyzed on average 13.9 PDs after the point of maximum senescence. Type I survivors exhibit short telomeres and strong hybridization at 5.2 kb and 6.7 kb, which is due to amplification of the tandemly repeated Y′ short and Y′ long elements, respectively. The telomeres of type II survivor are extended and very heterogeneous in size.

**Fig 5:**
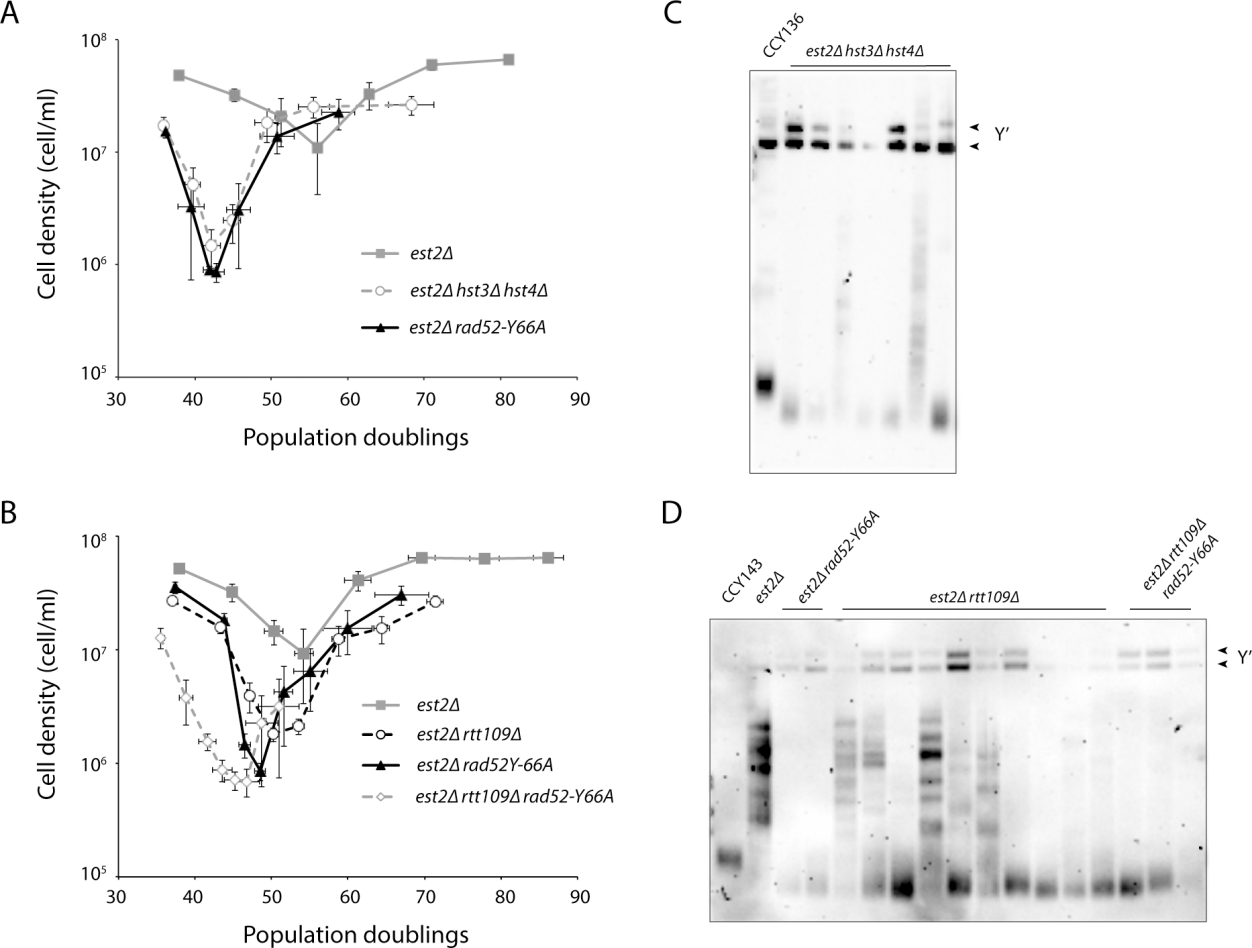
Hyper- and hypoacetylation of H3K56 causes accelerated senescence. (A, B) Senescence rates were measured in liquid culture by serial passaging of haploid meiotic progeny of the indicated genotypes, derived from the sporulation of CCY136 (A) and CCY143 (B). Mean ± SE for at least five independent spore isolates for each genotype is shown. (C, D) Telomere Southern blot of survivors of the indicated genotypes obtained from the liquid culture senescence assays from A and B. The parental diploids (CCY136 and CCY143) were included in the blots. *est2Δ hst3Δ hst4Δ* and *est2Δ rtt109Δ* survivors were analyzed on average 21.5 and 19.5 PDs, respectively, after the point of maximum senescence. Type I survivors exhibit short telomeres and strong hybridization at 5.2 kb and 6.7 kb, which is due to amplification of the tandemly repeated Y′ short and Y′ long elements, respectively. The telomeres of type II survivor are extended and very heterogeneous in size.

To further dissect the function of Rad52 at telomeres in the absence of telomerase, we used a specific class of *rad52* mutants (class C mutants, specifically *rad52-Y66A* and *rad52-R70A*) that are proficient for mitotic recombination but defective in DNA strand annealing and the repair of DSBs [30–32]. The efficiency of BIR is reduced only 2.7-fold in class C mutants [32], whereas *rad51*Δ mutants exhibit a ~140-fold reduction using the same assay [16], suggesting that class C mutants can perform Rad51-dependent BIR. We find that *est2Δ rad52-R70A* and *est2Δ rad52-Y66A* double mutants senesce faster than *est2Δ* single mutants (Fig 4A, *est2Δ* vs. *est2Δ rad52-R70A*, *p* < 10^−6^; Fig 4B, *est2Δ* vs. *est2Δ rad52-Y66A*, *p* = 0.003; Fig 4C, *est2Δ* vs. *est2Δ rad52-Y66A*, *p* = 0.001). Interestingly, survivors generated from *est2Δ rad52-R70A* and *est2Δ rad52-Y66A* strains are all type I (Fig 4D and Fig 5D). Since type II survivors grow better than type I survivors, survivors generated from a liquid culture senescence assay, as done here, should all be type II [52] unless the strain in question has a defect in forming type II survivors. In our experiments, 9 out of 9 survivors generated from *est2Δ* mutants, examined 6 population doublings (PDs) after they had recovered from the point of maximum senescence (for each *est2Δ* mutant, this means the first time point after the point of maximum senescence in the liquid culture senescence assays), were type II. Deleting *RAD51* in telomerase-null cells blocks the formation of type I survivors [12]. We find that *est2Δ rad51Δ rad52-Y66A* triple mutants cannot form any survivors (Fig 4B), supporting that the strand annealing activity of Rad52 is needed to perform Rad51-independent BIR and to form type II survivors.

The *rad52* class C mutants behave similarly to *rad59Δ*, which is also defective for Rad51-independent BIR and causes telomerase-negative strains to senesce fast and to be unable to form type II survivors [12, 17, 21]. We find that *est2Δ rad52-Y66A* senesces faster than *est2Δ rad59Δ* (*p* = 0.02), and deletion of *RAD59* does not enhance the senescence of *est2Δ rad52-Y66A* (Fig 4C), indicating that *rad52-Y66A* is epistatic to *rad59Δ* during senescence. However, *rad52-Y66A* has a greater effect on senescence than *rad59Δ*, suggesting that the accelerated senescence of telomerase-null *rad52* class C mutants is not solely due to a loss of Rad51-independent BIR. Interestingly, *est2Δ rad52-Y66A rad59Δ* triple mutants show a defect in the formation of survivors (Fig 4C and S3 Fig). Of the four *est2Δ rad52-Y66A rad59Δ* followed, three showed a prolonged delay before survivors arose and one never formed survivors at all during the duration of the experiment. This observation implies that, while Rad52 and Rad59 function in the same pathway during senescence, they have nonoverlapping functions with regard to survivor formation, and is consistent with other reports suggesting that Rad59 has Rad52-independent functions [53–55].

### Rad52 is not important for Rad5-dependent error-free post-replication repair at telomeres during senescence

Having established that Rad52 has non-BIR-related functions in preventing accelerated senescence, we asked whether Rad52 participates in error-free post-replication repair (PRR) at telomeres during senescence. Error-free PRR is thought to utilize the newly synthesized sister chromatid as a template for DNA synthesis to bypass DNA lesions (reviewed in [56]). It was shown that error-free PRR utilizes recombination proteins in the repair of MMS- and UV-induced DNA damage [57]. Rad5 is a key component of the error-free PRR pathway and absence of Rad5 accelerates senescence in a telomerase-negative strain [22]. We analyzed the rate of senescence of *est2Δ*, *est2Δ rad5Δ*, *est2Δ rad52-Y66A*, and *est2Δ rad5Δ rad52-Y66A* strains (S4 Fig). We find that *est2Δ rad5Δ* exhibits accelerated senescence (*est2Δ* vs. *est2Δ rad5Δ*, *p* = 0.001), as previously reported, and *est2Δ rad52-Y66A* senesces faster than *est2Δ rad5Δ* (*p* < 10^−6^). The *est2Δ rad5Δ rad52-Y66A* triple mutant senesces the fastest (*est2Δ rad52-Y66A* vs. *est2Δ rad5Δ rad52-Y66A*, *p* = 0.001), appearing to have combined the effects of *rad5Δ* and *rad52-Y66A* in an additive manner, suggesting that these mutations affect separate pathways. Consistent with our data, it has been previously reported that *rad5Δ rad52Δ* mutants exhibit a strong synthetic growth defect that is exacerbated in the absence of telomerase [22], and that Rad5 and Rad52 have independent functions during the bypass of thymine dimers [58]. These results indicate that Rad52-mediated HDR and Rad5-mediated error-free PRR act in at least partially non-overlapping pathways to prevent accelerated senescence.

### Defective SCR results in faster senescence and an inability to form type II survivors

There are several other possible non-BIR mechanisms through which Rad52 may prevent accelerated senescence, including recombination involving sister chromatids. *rad52* class C mutants have been previously reported to be defective in SCR [33]. This study also found that defective regulation of H3K56 acetylation also impairs SCR. H3K56 is acetylated by the histone acetyltransferase Rtt109 [59–61] and deacetylated by the histone deacetylases Hst3 and Hst4 [62, 63]. Both hyper-acetylation (e.g. *hst3Δ hst4Δ* and *H3K56Q* mutants) and hypo-acetylation of H3K56 (e.g. *rtt109Δ* and *H3K56R* mutants) decrease SCR [33]. We hypothesized that defective SCR may explain the senescence and survivor phenotype of *est2Δ rad52* class C mutants. If so, then mutants affecting H3K56 acetylation should behave similarly with respect to senescence and survivor formation. Consistent with this idea, both *est2Δ hst3Δ hst4Δ* and *est2Δ rtt109Δ* mutants exhibit accelerated senescence (*est2Δ* vs. *est2Δ hst3Δ hst4Δ*, *p* < 10^−6^; *est2Δ* vs. *est2Δ rtt109Δ*, *p* = 0.02) and are defective in type II survivor formation (Fig 5). However, unlike *est2Δ rad52* class C mutants, which do not form any type II survivors, *est2Δ hst3Δ hst4Δ* and *est2Δ rtt109Δ* mutants are defective, but still able to form type II survivors. As mentioned above, survivors generated from a liquid culture senescence assay are typically all type II. Two of seven *est2Δ hst3Δ hst4Δ* survivors (Fig 5C), and five of ten *est2Δ rtt109Δ* survivors (Fig 5D), were type II.

*hst3Δ hst4Δ rad52-Y66A* strains are synthetic lethal (S5A Fig), similar to *hst3Δ hst4Δ rad52Δ* [64]. In addition, *rtt109Δ rad52-Y66A* double mutants are synthetic sick (S5A Fig), similar to *rtt109Δ rad52Δ* double mutants [65]. These results indicate that while Rad52-dependent strand annealing and H3K56 acetylation are both important for SCR, to delay senescence, and for type II survivor formation, they function in different pathways. It is also possible that acetylation of H3K56 is important to delay senescence and promote type II survivor formation independently of its role in SCR.

## Discussion

The importance of Rad52 in delaying senescence and for telomerase-independent telomere maintenance in post-senescence survivors was first described over twenty years ago [9]. While much is now known about the role of HDR in telomerase-independent telomere maintenance in yeast as well as other organisms, including humans, the function of HDR during senescence is less-well understood. Rad52-mediated BIR has previously been implicated in preventing accelerated senescence, and it is thought that both Rad51-dependent and Rad51-independent BIR are involved [21–23]. In this study, we show that non-BIR functions of Rad52, involving recombination between sister chromatids, are also required to delay senescence. We also find that proper regulation of H3K56 acetylation is important in preventing accelerated senescence. We present a model where Rad52-mediated HDR mechanisms act at telomeres during telomere attrition-induced senescence.

We first tried to study the role of Rad52 during senescence with a telomere sequencing assay that has been used to detect telomere recombination events, or more specifically, intertelomeric recombination events and unequal sister telomere recombination events. Using this assay, it was estimated that such recombination events occur at a rate of 0.3% per telomere per generation [34]. Surprisingly, we find that the frequency of these events is not reduced by the deletion of *RAD52*—in fact, the frequency is even slightly increased (Fig 1). We show that a significant fraction of these events are caused by errors introduced during PCR amplification, propagation in *E*. *coli*, and/or DNA sequencing. Our results suggest that intertelomeric and unequal sister telomere recombination events occur at a substantially lower rate than 0.3% per telomere per generation. Furthermore, our findings indicate that data obtained previously with this assay suggesting the preferential recombination of short telomeres in senescing cells may need to be re-examined [37, 41], especially considering that telomere sequencing did not detect an increase in divergence events at an artificially-induced very short telomere [35]. However, it is known that Rad52 is important to act on this very short telomere to delay senescence and Rad52 is preferentially recruited to short telomeres in telomerase-negative cells [19, 35]. In addition, recombination intermediates accumulate as telomeres shorten in *tlc1Δ* cells [66]. Therefore, Rad52-mediated HDR does preferentially act at short telomeres. It is important to note that our findings do not invalidate the use of the telomere sequencing assay to assess telomere recombination, but one needs to keep in mind that there is a background level of sequence divergence events that are not a result of recombination in vivo. Our data also allow us to conclude that these sequence divergence events are unrelated to the function of Rad52 during replicative senescence. It is formally possible that in the presence of Rad52, sequence divergence events (that are not due to technical artefacts) are the result of Rad52-mediated recombination events, while in the absence of Rad52, the divergence events are the result of Rad52-independent mechanisms. However, we find this possibility unlikely given the remarkably similar telomere sequence divergence profiles of *est2Δ* and *est2Δ rad52* strains.

**Fig 6:**
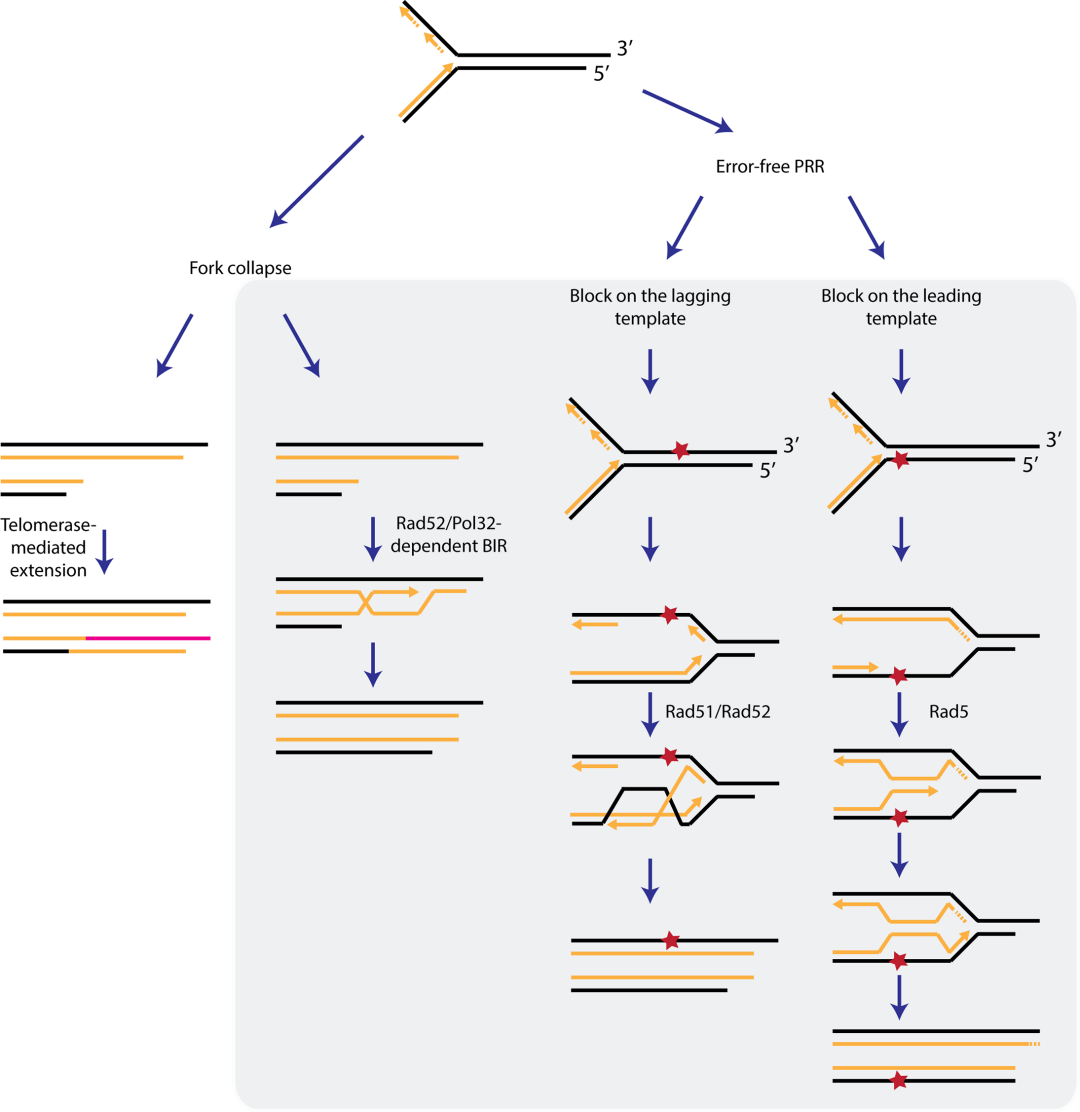
A model to describe the known Rad52-mediated mechanisms that function at telomeres during replicative senescence. Replication forks traveling through subtelomeric and telomeric sequences frequently encounter impediments to their progression. A fork collapse would leave a truncated telomere, which can be elongated by telomerase (far left). In the absence of telomerase (gray box), the truncated telomere can be repaired by BIR. Fork impediments can be dealt with via error-free PRR. Blocks of lagging and leading strand synthesis can be repaired via Rad5-independent and Rad5-dependent error-free PRR, respectively.

Replication forks have difficulty progressing through subtelomeric and telomeric sequences, causing forks to stall and collapse [67, 68]. A collapsed replication fork at a telomere would lead to a truncated telomere, and it has previously been shown that such truncations do occur in vivo, and that they are rapidly extended by telomerase [49]. In the absence of telomerase, telomere truncation events are likely repaired by BIR using the untruncated sister telomere as a template (Fig 6).

Eliminating BIR by the deletion of either *POL32* or *RAD52* in telomerase-null cells leads to a similar phenotype: accelerated senescence and an inability to form survivors [9, 10]. However, our data show that deletion of *RAD52* in the absence of telomerase is more severe than deletion of *POL32*, suggesting that Rad52 has functions in addition to BIR during senescence (Fig 3). Double-strand break repair (DSBR), synthesis-dependent strand annealing (SDSA), and single-strand annealing (SSA) are all well-studied Rad52-mediated HDR mechanisms, but all of these are initiated to repair a two-ended DSB. In the absence of exogenous stress, most DSBs occur during DNA replication, likely via replication fork collapse. A fork collapse would lead to a one-ended DSB, which is converted to a two-ended DSB when a replication fork coming from the opposite direction reaches the site of the fork collapse. However, a replication fork that collapses while traversing a chromosome end would stay one-ended since there is no replication fork coming from the distal end of the telomere. The one-ended DSB can be repaired by BIR, but not DSBR, SDSA, or SSA. Thus, Rad52-mediated DSBR, SDSA, or SSA are unlikely to be involved in delaying senescence.

We find that *hst3Δ hst4Δ* and *rtt109Δ* mutants, which cause hyper- and hypo-acetylation of H3K56, respectively, also display rapid senescence in the absence of telomerase (Fig 5). Like *rad52* class C mutants, *hst3Δ hst4Δ* and *rtt109Δ* strains have defects in SCR [33]. Thus, we hypothesize that, in addition to its function in BIR, Rad52 delays senescence through a mechanism involving recombination of sister chromatids. Error-free PRR utilizes a newly synthesized sister chromatid as a template to replicate past replication fork impediments, so it could be seen as a type of SCR. Rad52-mediated HDR activity has also been implicated in error-free PRR, in both a Rad5-dependent and a Rad5-independent manner [57, 58]. Rad5 localizes to a subset of telomeres during S and G2 phases, even in the absence of exogenous stress, and deletion of *RAD5* in telomerase-null cells leads to accelerated senescence [22]. However, we find that *rad52-Y66A*, which is still proficient in Rad51-dependent BIR, and *rad5Δ* have additive effects in terms of telomere attrition-induced senescence (S4 Fig), suggesting that if Rad52 participates in error-free PRR to delay senescence, it does so via a mechanism separate from Rad5-dependent error-free PRR. Consistent with this view, we have reported that the Shu complex, which is required for efficient HDR and involved in Rad5-mediated error-free PRR [57], is not important for delaying senescence [69]. It has been suggested that error-free PRR proceeds via a Rad5-mediated pathway when the lesion is on the leading strand template, and a Rad52-mediated pathway when the lesion is on the lagging strand template [58]. We propose that this situation may be occurring at telomeres during senescence (Fig 6).

We believe that replication problems at chromosome ends are amplified as telomeres get shorter. *est2Δ rad52Δ* cells do not exhibit a growth defect immediately after the loss of telomerase (S6 Fig), indicating that telomeres need to shorten before Rad52 becomes important. As mentioned above, Rad52 and recombination intermediates accumulate at telomeres as they shorten [19, 66]. One possible explanation for increased replication problems at short telomeres is that telomere shortening triggers TERRA transcription [70], which could impede replication because of the replication fork encountering either the RNA polymerase machinery or RNA-DNA hybrids. Increased TERRA transcription and telomeric RNA-DNA hybrids both stimulate recombination at telomeres [39, 40]. Mammalian RAD51 and BRCA2, which performs many of the functions of yeast Rad52 [71], are also required for proper telomere maintenance [72, 73], indicating that the importance of HDR at telomeres is highly conserved throughout evolution.

## Materials and Methods

### Yeast media, strains, and plasmids

Standard yeast media and growth conditions were used [74, 75]. All yeast strains used in this study are *RAD5* derivatives of W303 [76, 77] and are listed in Table 1. Telomeres of 166 bp, 213 bp, and 230 bp, amplified by telomere PCR, were cloned into the pCR-Blunt vector from the Zero Blunt PCR Cloning Kit (Invitrogen) to generate plasmids pCC3, pCC6, and pCC2, respectively. pCC3 and pCC2 were cut with *Eco*RI, and the telomere-containing fragment in each was subcloned into *Eco*RI-cut pRS306 (ATCC) to generate plasmids pCC9 and pCC10 (two isolates containing 166 bp-long telomere sequences), and pCC7 and pCC8 (two isolates containing 230 bp-long telomere sequences). pCC9, pCC10, pCC7, and pCC8 were cut with *Nco*I and transformed into yeast strain W9100-12C to make CCY36, CCY37, CCY34, and CCY35, respectively. *RAD52* was then deleted in CCY36 and CCY35 to generate CCY47 and CCY46, respectively.

**Table 1:** List of *S*. *cerevisiae* strains used in this study. All strains are *ade2-1 his3-11*,*15 leu2-3*,*112 trp1-1 ura3-1 RAD5* unless indicated otherwise.

Strain	Relevant genotype	Source
CCY16	*MAT***a***/*α *est2ΔURA3/EST2 rad52ΔkanMX/RAD52*	This study
CCY18	*MAT***a***/*α *est2ΔURA3/EST2 pol32ΔkanMX/POL32*	This study
CCY8	*MAT***a***/*α *est2ΔURA3/EST2 rad59ΔkanMX/RAD59*	This study
CCY101	*MAT***a***/*α *est2ΔkanMX/EST2 RAD52-RFP/RAD52-RFP CDC13-YFP/CDC13-YFP RAP1-CFP*::*LEU2/RAP1-CFP*::*LEU2 pol32ΔnatMX/POL32*	This study
CCY36	*MAT*α *ura3-1*::*TEL6R(166 bp)-URA3*	This study
CCY37	*MAT*α *ura3-1*::*TEL6R(166 bp)-URA3*	This study
CCY34	*MAT*α *ura3-1*::*TEL6R(230 bp)-URA3*	This study
CCY35	*MAT*α *ura3-1*::*TEL6R(230 bp)-URA3*	This study
CCY47	*MAT*α *rad52ΔkanMX ura3-1*::*TEL6R(166 bp)-URA3*	This study
CCY46	*MAT*α *rad52ΔkanMX ura3-1*::*TEL6R(230 bp)-URA3*	This study
MCY713	*MAT***a***/*α *rad52ΔkanMX/RAD52 ura3-1*::*tel6R(166 bp)-URA3/ura3-1 ade2-1/ade2-1*	This study
MCY712	*MAT***a***/*α *rad52ΔkanMX/RAD52 ura3-1*::*tel6R(230 bp)-URA3/ura3-1 ade2-1/ade2-1*	This study
W8758	MAT**a**/α *rad52Δ*::*HIS3/RAD52 lys2/LYS2 trp1/TRP1 ADE2/ADE2 bar1*::*LEU2/bar1*::*LEU2 RAD5/RAD5*	Rothstein lab collection
CCY155	*MAT***a***/*α *est2ΔURA3/EST2 pol32ΔnatMX/POL32 rad52ΔkanMX/RAD52*	This study
CCY114	*MAT***a***/*α *est2ΔURA3/EST2 rad52-R70A/RAD52*	This study
CCY126	*MAT***a***/*α *est2ΔURA3/EST2 rad51ΔkanMX/RAD51 rad52-Y66A/RAD52*	This study
CCY127	*MAT***a***/*α *est2ΔURA3/EST2 rad52-Y66A/RAD52 rad59ΔkanMX/RAD59*	This study
CCY159	*MAT***a***/*α *est2ΔURA3/EST2 rad5ΔkanMX/RAD5 rad52-Y66A/RAD52*	This study
CCY136	*MAT***a***/*α *est2ΔURA3/EST2 hst3ΔkanMX/HST3 hst4ΔnatMX/HST4 rad52-Y66A/RAD52*	This study
CCY143	*MAT***a***/*α *est2ΔURA3/EST2 rtt109ΔkanMX/RTT109 rad52-Y66A/RAD52*	This study
MCY661	*MAT***a***/*α *est2ΔURA3/EST2 rad24ΔnatMX/RAD24 rad52ΔkanMX/RAD52*	This study
W9100-12C	*MAT*α *ADE2*	[78]

### Telomere PCR and sequencing

Telomere VI-R was amplified by PCR using Phusion High-Fidelity DNA Polymerase (New England Biolabs), essentially as previously described [49]. Telomere PCR products were purified using a QIAquick Gel Extraction Kit (Qiagen), cloned using a Zero Blunt PCR Cloning Kit or a Zero Blunt TOPO PCR Cloning Kit (Invitrogen), and transformed into One Shot TOP10 Chemically Competent *E*. *coli* (Invitrogen). Individual clones were sequenced by GATC Biotech (except for Fig 2, where sequencing was performed by GENEWIZ), and the resulting data were analyzed using Sequencher software (Gene Codes). The Sequencher files are included as Supporting Information. Excel files recording telomere sequence divergence data are included as S1 Dataset (for Fig 1) and S2 Dataset (for Fig 2). For each set of sequences, the longest telomere without divergent sequence was used as a reference telomere to which all other telomeres are compared to determine whether divergence has occurred. A sequence was determined to be non-divergent if it matches perfectly to the consensus, if it contains single point mutations, or if it contains insertions or deletions of 6 nucleotides or less.

### Fluorescence microscopy

Cells used for live-cell imaging were cultured in synthetic complete media. Microscopy was performed using a DeltaVision Deconvolution Microscope (Applied Precision) with InsightSSI, an Olympus UPLS Apo 100x oil objective with 1.4 numerical aperture, and a CoolSNAP HQ^2^ camera.

### Senescence assay

Liquid culture senescence assays were performed as previously described [37, 79]. All senescence assays started with the sporulation of *est2Δ*/*EST2* heterozygous diploids. With the exception of S3 Fig, senescence data were plotted with PDs on the x-axis, not time (i.e. days), because telomere shortening is a function of cell division and not time. Moreover, using PDs as a metric prevents slow growth associated with a particular mutation to be mistakenly interpreted as having an effect on senescence. For clarity, telomerase-positive control strains for each experiment are shown in separate graphs (S5B and S7 Figs). We performed an unpaired two-tailed *t*-test to evaluate the difference in PDs at maximum senescence between two strains.

### Telomere Southern blot

Genomic DNA was isolated using a Wizard Genomic DNA Purification Kit (Promega), digested with *Xho*I restriction endonuclease, separated by agarose gel electrophoresis, transferred to a Hybond-N^+^ membrane (GE Healthcare), and hybridized to a telomere-specific (5′-CACCACACCCACACACCACACCCACA-3′) digoxigenin-labeled probe.

## Supporting Information

S1 Fig Spontaneous Rad52 focus formation is increased in *pol32Δ* and *est2Δ pol32Δ* mutants. (A) Logarithmically growing cells expressing Rad52-RFP, derived from the sporulation of CCY101, were visualized by fluorescence microscopy approximately 35 population doublings after the isolation of haploid spores. (B) The percentage of cells with Rad52 foci was determined for the indicated strains. P-values were calculated using Fisher’s exact test, p = 5 x 10^−4^ (**) and p = 1 x 10^−2^ (*).
(PDF)

S2 Fig Measuring sequence divergence after PCR amplification, cloning, and sequencing of telomere sequences of defined length. Two plasmids containing telomere sequences, 166 bp and 213 bp in length, were amplified by PCR, re-cloned into the same vector, and sequenced. Two telomeres, 166 bp and 230 bp in length, were also inserted at the *URA3* locus in wild-type and *rad52Δ* strains. The resulting strains were clonally propagated for ~30 population doublings, and the inserted telomeres were then amplified by PCR, cloned, and sequenced. Sequence divergence was determined using the same rules as for the identification of divergent sequences amplified from a specific telomere (i.e. telomere VI-R) from a population of cells (see Materials and Methods).
(PDF)

S3 Fig *est2Δ rad52-Y66A rad59Δ* mutants are delayed in forming survivors. The graph is the same as Fig 4C, except that the data are plotted in ‘Days’ on the x-axis instead of ‘Population Doublings’. In addition, the independent *est2Δ rad52-Y66A rad59Δ* replicates are plotted instead to highlight the delay/defect in survivor formation.
(PDF)

S4 Fig Rad52 and Rad5 function in separate pathways to delay senescence. Senescence rates were measured by serial passaging of *est2Δ* (n = 6), *est2Δ rad5Δ* (n = 16), *est2Δ rad52-Y66A* (n = 11), and *est2Δ rad5Δ rad52-Y66A* strains (n = 8), derived from the sporulation of CCY159, in liquid culture. Cell density was measured each day after 24 h of growth in liquid culture, followed by dilution to 2 x 10^5^ cells/ml. Mean ± SE for each genotype is shown.
(PDF)

S5 Fig Hyper- and hypo-acetylation of H3K56 causes synthetic lethality and sickness, respectively, with *rad52-Y66A*. (A) Representative tetrads derived from the sporulation of CCY136 and CCY143 are shown. Colony sizes were measured for each genotype and normalized to wild type. Mean ± SE is shown. (B) Graphs are identical to the ones shown in Fig 5, except that telomerase-positive control strains have been included (2 to 4 isolates each).
(PDF)

S6 Fig Deletion of *RAD52* does not affect cell growth early after the loss of telomerase. (A) Representative tetrads derived from the sporulation of CCY16 are shown. (B) Colony sizes were measured for each genotype and normalized to wild type. Mean ± SE is shown.
(PDF)

S7 Fig Senescence data with telomerase-positive control strains included. Graphs are identical to the ones shown in the main figures, except telomerase-positive control strains have been included (1 to 4 isolates each).
(PDF)

S1 Sequencher File Telomere sequence analysis of *est2Δ* for Fig 1. (SPF)

S2 Sequencher File Telomere sequence analysis of *est2Δ rad52Δ* and *est2Δ rad59Δ* for Fig 1. (SPF)

S3 Sequencher File Telomere sequence analysis of *est2Δ pol32Δ* for Fig 1. (SPF)

S4 Sequencher File Sequence analysis of plasmid controls for S2 Fig. (SPF)

S5 Sequencher File Sequence analysis of integrated-telomere controls in wild type for S2 Fig. (SPF)

S6 Sequencher File Sequence analysis of integrated-telomere (166 bp) controls in *rad52Δ* for S2 Fig. (SPF)

S7 Sequencher File Sequence analysis of integrated-telomere (230 bp) controls in *rad52Δ* for S2 Fig. (SPF)

S8 Sequencher File Telomere sequence analysis for Fig 2. (SPF)

S1 Dataset Telomere sequence divergence data for Fig 1. (XLSX)

S2 Dataset Telomere sequence divergence data for Fig 2. (XLSX)
