# Supplementary material for: Multiple Rad52-Mediated Homology-Directed Repair Mechanisms Are Required to Prevent Telomere Attrition-Induced Senescence in Saccharomyces cerevisiae
Source: PLoS Genet. 2016 Jul 18;12(7):e1006176. doi: 10.1371/journal.pgen.1006176 (PMC4948829; doi:10.1371/journal.pgen.1006176)
Supplement: S1 Fig — (A) Logarithmically growing cells expressing Rad52-RFP, derived from the sporulation of CCY101, were visualized by fluorescence microscopy approximately 35 population doublings after the isolation of haploid spores. (B) The percentage of cells with Rad52 foci was determined for the indicated strains. P-values were calculated using Fisher’s exact test, p = 5 x 10^−4^ (**) and p = 1 x 10^−2^ (*). (PDF) [file pgen.1006176.s001.pdf]

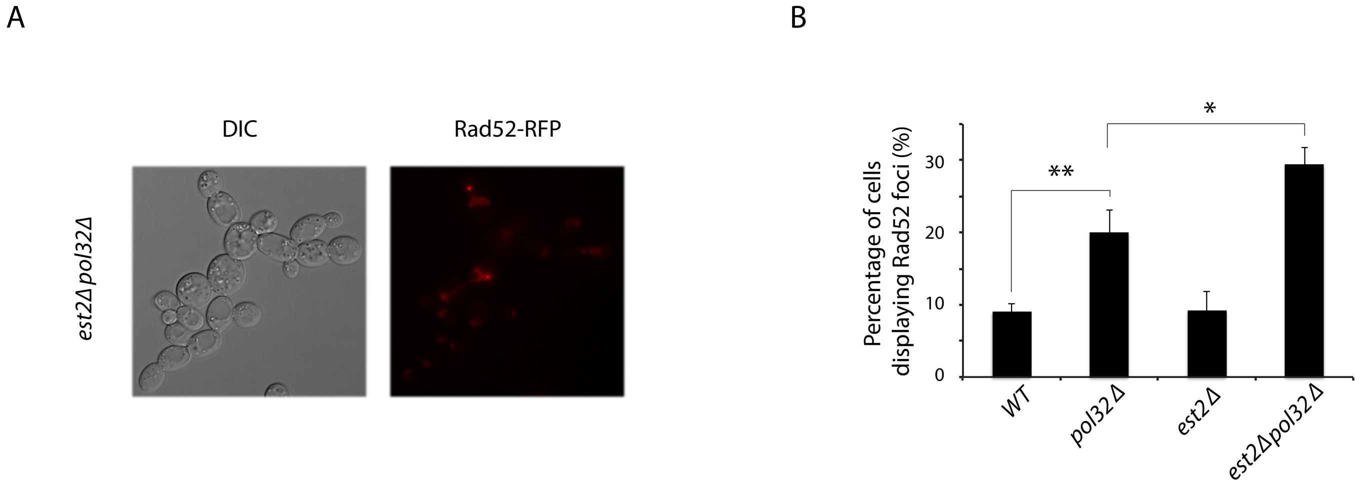

Figure S1. Spontaneous Rad52 focus formation is increased in *pol32Δ* and *est2Δ pol32Δ* mutants. (A) Logarithmically growing cells expressing Rad52-RFP, derived from the sporulation of CCY101, were visualized by fluorescence microscopy approximately 35 population doublings after the isolation of haploid spores. (B) The percentage of cells with Rad52 foci was determined for the indicated strains. P-values were calculated using Fisher's exact test, $p = 5 \times 10^{-4}$ (**) and $p = 1 \times 10^{-2}$ (*).
