# Supplementary material for: Multiple Rad52-Mediated Homology-Directed Repair Mechanisms Are Required to Prevent Telomere Attrition-Induced Senescence in Saccharomyces cerevisiae
Source: PLoS Genet. 2016 Jul 18;12(7):e1006176. doi: 10.1371/journal.pgen.1006176 (PMC4948829; doi:10.1371/journal.pgen.1006176)
Supplement: S2 Fig — Two plasmids containing telomere sequences, 166 bp and 213 bp in length, were amplified by PCR, re-cloned into the same vector, and sequenced. Two telomeres, 166 bp and 230 bp in length, were also inserted at the URA3 locus in wild-type and rad52Δ strains. The resulting strains were clonally propagated for ~30 population doublings, and the inserted telomeres were then amplified by PCR, cloned, and sequenced. Sequence divergence was determined using the same rules as for the identification of divergent sequences amplified from a specific telomere (i.e. telomere VI-R) from a population of cells (see Materials and Methods). (PDF) [file pgen.1006176.s002.pdf]

		Telomere length (bp)	Clones sequenced	Divergent sequences	Percent
Plasmid cloned	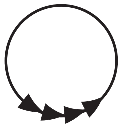	166	67	3	4.5
		213	63	2	3.2
		166 + 213	130	5	3.8
Internally integrated in WT	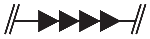	166	123	5	4.1
		230	138	6	4.3
		166 + 230	261	11	4.2
Internally integrated in *rad52Δ* mutants	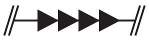	166	175	13	7.4
		230	169	12	7.1
		166 + 230	344	25	7.3

Figure S2. Measuring sequence divergence after PCR amplification, cloning and sequencing of telomere sequences of defined length. Two plasmids containing telomeres sequences, 166 bp and 213 bp in length, were amplified by PCR, re-cloned into the same vector, and sequenced. Two telomeres, 166 bp and 230 bp in length, were also inserted at the *URA3* locus in wild-type and *rad52Δ* strains. The resulting strains were clonally propagated for ~30 population doublings, and the inserted telomeres were then amplified by PCR, cloned, and sequenced. Sequence divergence was determined using the same rules for the identification of divergent sequences amplified from a specific telomere (i.e. telomere VI-R) from a population of cells (see Materials and Methods).
