# Supplementary material for: Multiple Rad52-Mediated Homology-Directed Repair Mechanisms Are Required to Prevent Telomere Attrition-Induced Senescence in Saccharomyces cerevisiae
Source: PLoS Genet. 2016 Jul 18;12(7):e1006176. doi: 10.1371/journal.pgen.1006176 (PMC4948829; doi:10.1371/journal.pgen.1006176)
Supplement: S3 Fig — The graph is the same as Fig 4C, except that the data are plotted in ‘Days’ on the x-axis instead of ‘Population Doublings’. In addition, the independent est2Δ rad52-Y66A rad59Δ replicates are plotted instead to highlight the delay/defect in survivor formation. (PDF) [file pgen.1006176.s003.pdf]

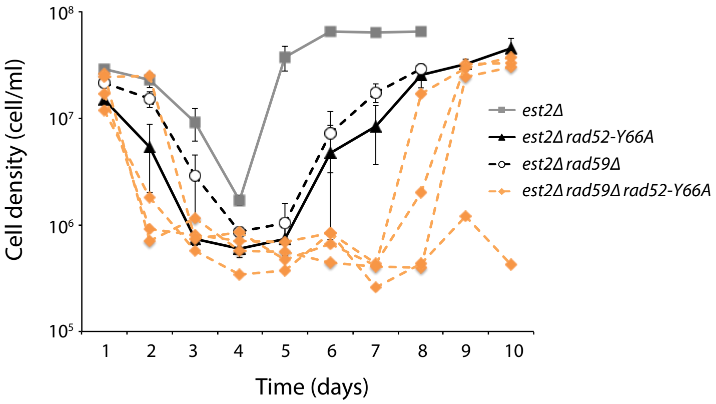

Figure S3. *est2Δ rad52-Y66A rad59Δ* mutants are delayed in forming survivors. The graph is the same as Figure 4C, except that the data are plotted in "Days" on the x-axis instead of "Population Doublings". In addition, the independent *est2Δ rad52-Y66A rad59Δ* replicates are plotted instead to highlight the delay/defect in survivor formation.
