# Supplementary material for: Multiple Rad52-Mediated Homology-Directed Repair Mechanisms Are Required to Prevent Telomere Attrition-Induced Senescence in Saccharomyces cerevisiae
Source: PLoS Genet. 2016 Jul 18;12(7):e1006176. doi: 10.1371/journal.pgen.1006176 (PMC4948829; doi:10.1371/journal.pgen.1006176)
Supplement: S4 Fig — Senescence rates were measured by serial passaging of est2Δ (n = 6), est2Δ rad5Δ (n = 16), est2Δ rad52-Y66A (n = 11), and est2Δ rad5Δ rad52-Y66A strains (n = 8), derived from the sporulation of CCY159, in liquid culture. Cell density was measured each day after 24 h of growth in liquid culture, followed by dilution to 2 x 10^5^ cells/ml. Mean ± SE for each genotype is shown. (PDF) [file pgen.1006176.s004.pdf]

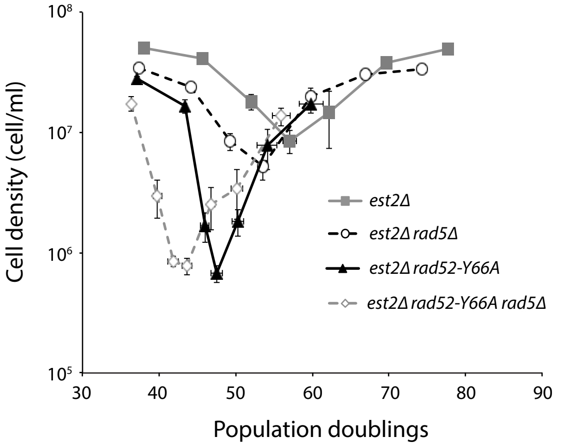

Figure S4. Rad52 and Rad5 function in separate pathways to delay senescence. Senescence rates were measured by serial passaging of *est2Δ* (n = 6), *est2Δ rad5Δ* (n = 16), *est2Δ rad52-Y66A* (n = 11) and *est2Δ rad5Δ rad52-Y66A* (n = 8) strains, derived from the sporulation of CCY159, in liquid culture. Cell density was measured each day after 24 h of growth in liquid culture, followed by dilution to 2×10^5 cells/ml. Mean ± SE for each genotype is shown.
