# Supplementary material for: Multiple Rad52-Mediated Homology-Directed Repair Mechanisms Are Required to Prevent Telomere Attrition-Induced Senescence in Saccharomyces cerevisiae
Source: PLoS Genet. 2016 Jul 18;12(7):e1006176. doi: 10.1371/journal.pgen.1006176 (PMC4948829; doi:10.1371/journal.pgen.1006176)
Supplement: S5 Fig — (A) Representative tetrads derived from the sporulation of CCY136 and CCY143 are shown. Colony sizes were measured for each genotype and normalized to wild type. Mean ± SE is shown. (B) Graphs are identical to the ones shown in Fig 5, except that telomerase-positive control strains have been included (2 to 4 isolates each). (PDF) [file pgen.1006176.s005.pdf]

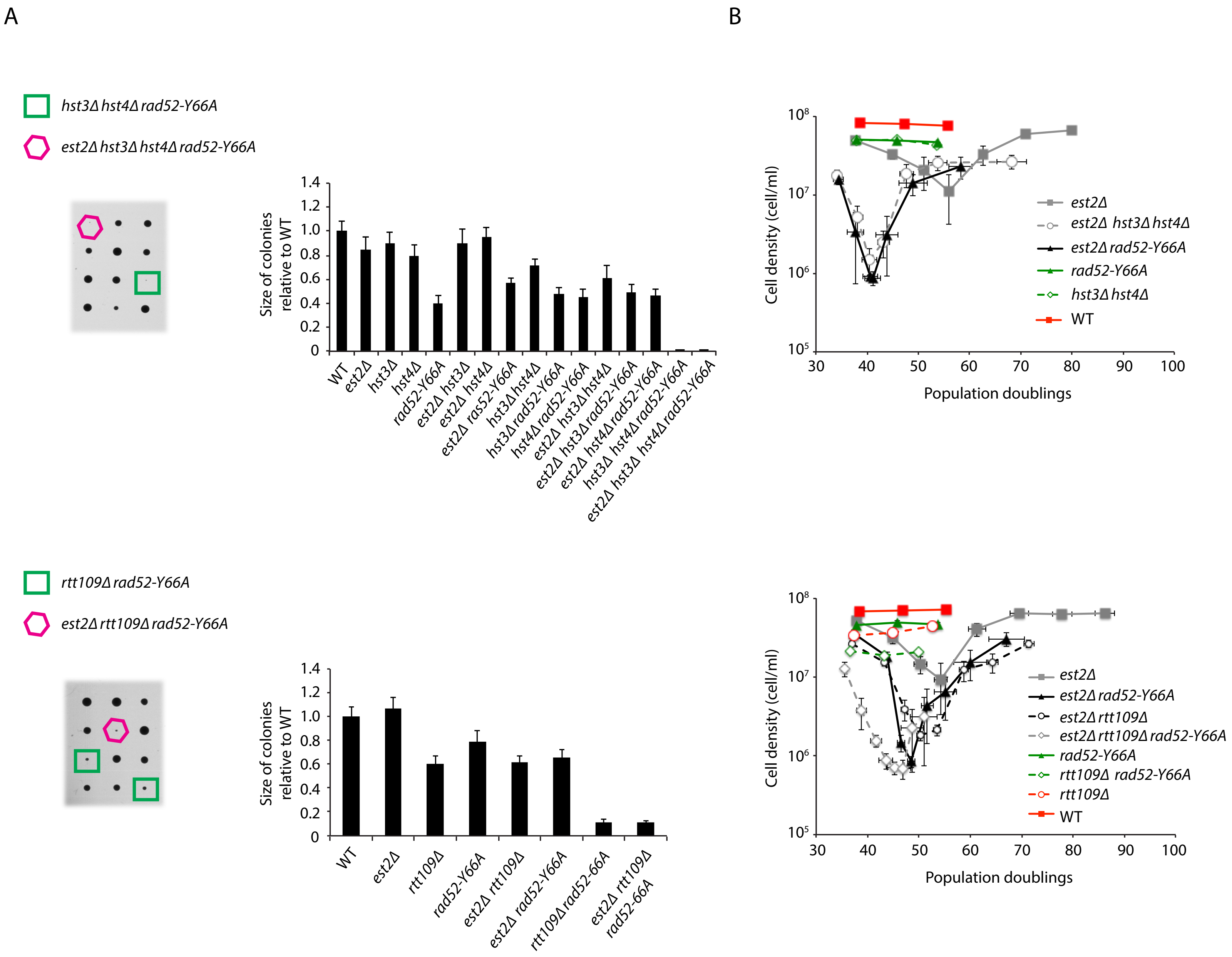

Figure S5. Hyper- and hypo-acetylation of H3K56 causes synthetic lethality and sickness, respectively, with *rad52-Y66A*. (A) Representative tetrads derived from the sporulation of CCY136 and CCY143 are shown. Colony sizes were measured for each genotype and normalized to wild type. Mean ± SE is shown. (B) Graphs are identical to the ones shown in Figure 5, except that telomerase-positive control strains have been included (2 to 4 isolates each).
