# Supplementary material for: Multiple Rad52-Mediated Homology-Directed Repair Mechanisms Are Required to Prevent Telomere Attrition-Induced Senescence in Saccharomyces cerevisiae
Source: PLoS Genet. 2016 Jul 18;12(7):e1006176. doi: 10.1371/journal.pgen.1006176 (PMC4948829; doi:10.1371/journal.pgen.1006176)
Supplement: S6 Fig — (A) Representative tetrads derived from the sporulation of CCY16 are shown. (B) Colony sizes were measured for each genotype and normalized to wild type. Mean ± SE is shown. (PDF) [file pgen.1006176.s006.pdf]

A

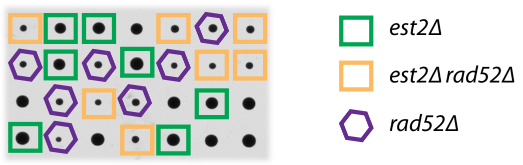

B

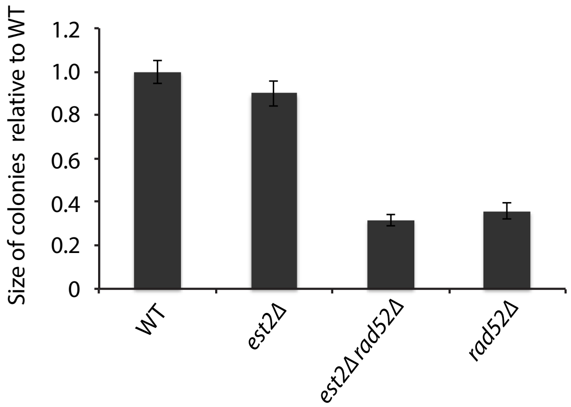

Figure S6. Deletion of *RAD52* does not affect cell growth early after the loss of telomerase. (A) Representative tetrads derived from the sporulation of CCY16 are shown. (B) Colony sizes were measured for each genotype and normalized to wild type. Mean ± SE is shown.
