# Supplementary material for: Multiple Rad52-Mediated Homology-Directed Repair Mechanisms Are Required to Prevent Telomere Attrition-Induced Senescence in Saccharomyces cerevisiae
Source: PLoS Genet. 2016 Jul 18;12(7):e1006176. doi: 10.1371/journal.pgen.1006176 (PMC4948829; doi:10.1371/journal.pgen.1006176)
Supplement: S7 Fig — Graphs are identical to the ones shown in the main figures, except telomerase-positive control strains have been included (1 to 4 isolates each). (PDF) [file pgen.1006176.s007.pdf]

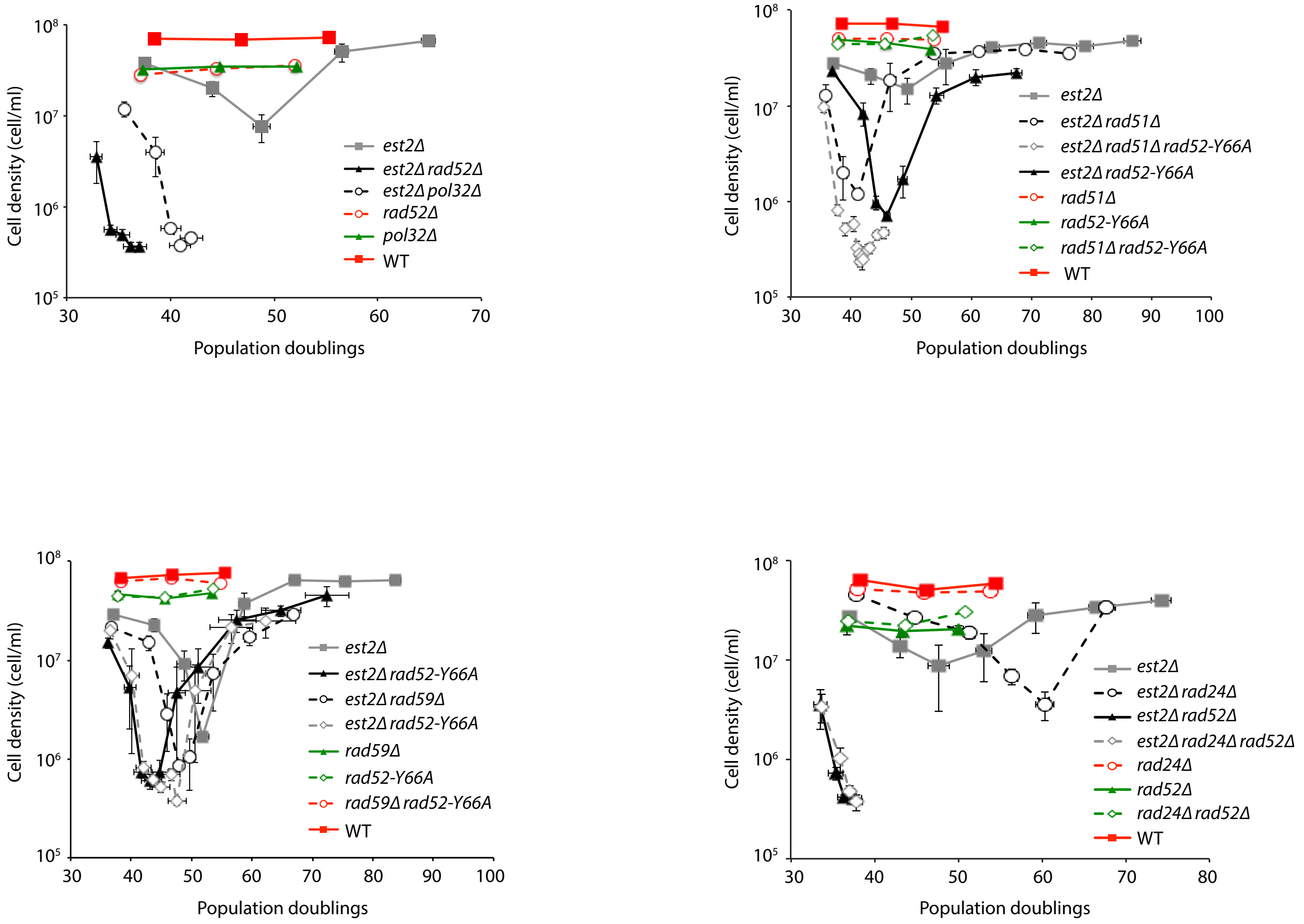

Figure S7. Senescence data with telomerase-positive control strains included. Graphs are identical to the ones shown in the main figures, except telomerase-positive control strains have been included (1 to 4 isolates each).
